# Novel syringe handle for one-handed anterior chamber paracentesis

**DOI:** 10.1186/s12886-025-04503-z

**Published:** 2025-11-28

**Authors:** Liangbo Linus Shen, Jay M. Stewart, Mitchel A. Cole, Madelynn E. Mackenzie, Frank Brodie

**Affiliations:** 1 Department of Ophthalmology, University of California, San Francisco, 490 Illinois Street, Floor 5, San Francisco, CA 94143-4081 USA; 2 Department of Ophthalmology, Duke University, 2351 Erwin Rd, Durham, NC 27705 USA

**Keywords:** Anterior chamber paracentesis, New instrument, Syringe

## Abstract

**Background:**

Anterior chamber (AC) paracentesis is a valuable diagnostic and therapeutic ophthalmic procedure, but the conventional technique poses ergonomic and safety challenges.

**Methods:**

We developed a custom, low-cost, 3D-printed syringe handle that enables one-handed AC paracentesis using standard 1-mL syringes by allowing single-finger plunger retraction. In a randomized controlled wet-lab study, ten ophthalmology residents each performed four AC paracentesis procedures (two with the novel device, two with the conventional technique) on porcine eyes. The primary outcome was successful aspiration of ≥ 100 µL of aqueous humor. Secondary outcomes included intraocular injury, aspirated volume, procedure time, and responses to an anonymous post-procedure survey.

**Results:**

All AC paracentesis procedures achieved ≥ 100 µL of fluid. No intraocular injuries occurred with the novel device, compared to 2 intraocular tissue contacts with the conventional method. The median aspirated volume was 120 µL (interquartile range [IQR]: 110–150 µL) with the novel device, close to the 100 µL threshold, with no extractions exceeding 200 µL. In contrast, the conventional method yielded a significantly higher median volume of 200 µL (IQR 140–270 µL; *P* < 0.001), with 25% of procedures exceeding 250 µL. The median procedure time was 26.9 s with the novel device versus 35.7 s with the conventional method, a 8.8 s (25%) reduction (*P* = 0.13). Participants generally preferred the novel device for ease of use, control, and perceived safety. Nine of 10 participants reported feeling more or much more confident about performing AC paracentesis safely with the novel syringe handle than with the conventional technique.

**Conclusions:**

We developed a novel syringe handle for one-handed AC paracentesis, which improved the safety and precision of the procedure while reducing procedural time. Its low-cost, reusable design and compatibility with standard syringes make it a practical tool for the procedure. Further clinical studies are warranted to explore broader applications.

**Supplementary Information:**

The online version contains supplementary material available at 10.1186/s12886-025-04503-z.

## Background

Anterior chamber (AC) paracentesis is a routine in-office ophthalmic procedure with important diagnostic and therapeutic applications. It involves inserting a fine needle into the anterior chamber to collect aqueous humor [1]. Clinically, it is used for diagnosing uveitis, endophthalmitis, or lymphoma and for rapidly lowering intraocular pressure in glaucoma or perioperatively for intravitreal injections [1–4]. Though generally safe, complications such as trauma to the cornea, iris, or lens, hyphema, keratitis, and endophthalmitis have been reported [1, 5, 6]. Precision and safety are therefore essential.

Conventional AC paracentesis techniques pose ergonomic and safety challenges. Passive aspiration by removing the syringe plunger offers no control over flow rate and makes fluid transfer for testing difficult. Sudden egress can lead to unpredictable intraocular pressure drops, AC shallowing, and complications such as gas entering the AC after pneumatic retinopexy or iris plugging the needle. Alternatively, using both hands—one to hold the syringe, the other to pull the plunger—limits the ability to stabilize the globe or adjust the slit lamp. This often forces the operator to look away from the needle to check volume, increasing the risk of over-aspiration or injury if the patient moves. A third method involves an assistant pulling the plunger while the ophthalmologist inserts the needle with one hand while using the other hand to stabilize the eye or to adjust the slit lamp, but this requires additional personnel and can result in inconsistent aspiration force, collapsed anterior chamber, or unintended trauma.

Previous efforts have aimed to improve safety and ease of use. The aqueous pipette, introduced 20 years ago, allows one-handed aspiration with a squeeze bulb [7]. However, it requires users to adapt to a new tool, and the extracted fluid can return to the chamber, which has limited its adoption in clinics. More recently, a plungerless AC paracentesis technique using an adhesive film dressing and a vacutainer was described, but passive collection may yield insufficient volumes, fluid extraction speed cannot be controlled, and vacutainers are not universally accessible [8].

To address these limitations, we developed a 3D-printed syringe handle that allows one-handed AC paracentesis using standard syringes. This snap-on device enables plunger retraction with a finger while the other hand stabilizes the globe. We evaluated its performance against the conventional technique in a randomized controlled wet-lab study using porcine eyes, followed by a post-procedure survey.

## Methods

### Syringe handle design

**Fig. 1:**
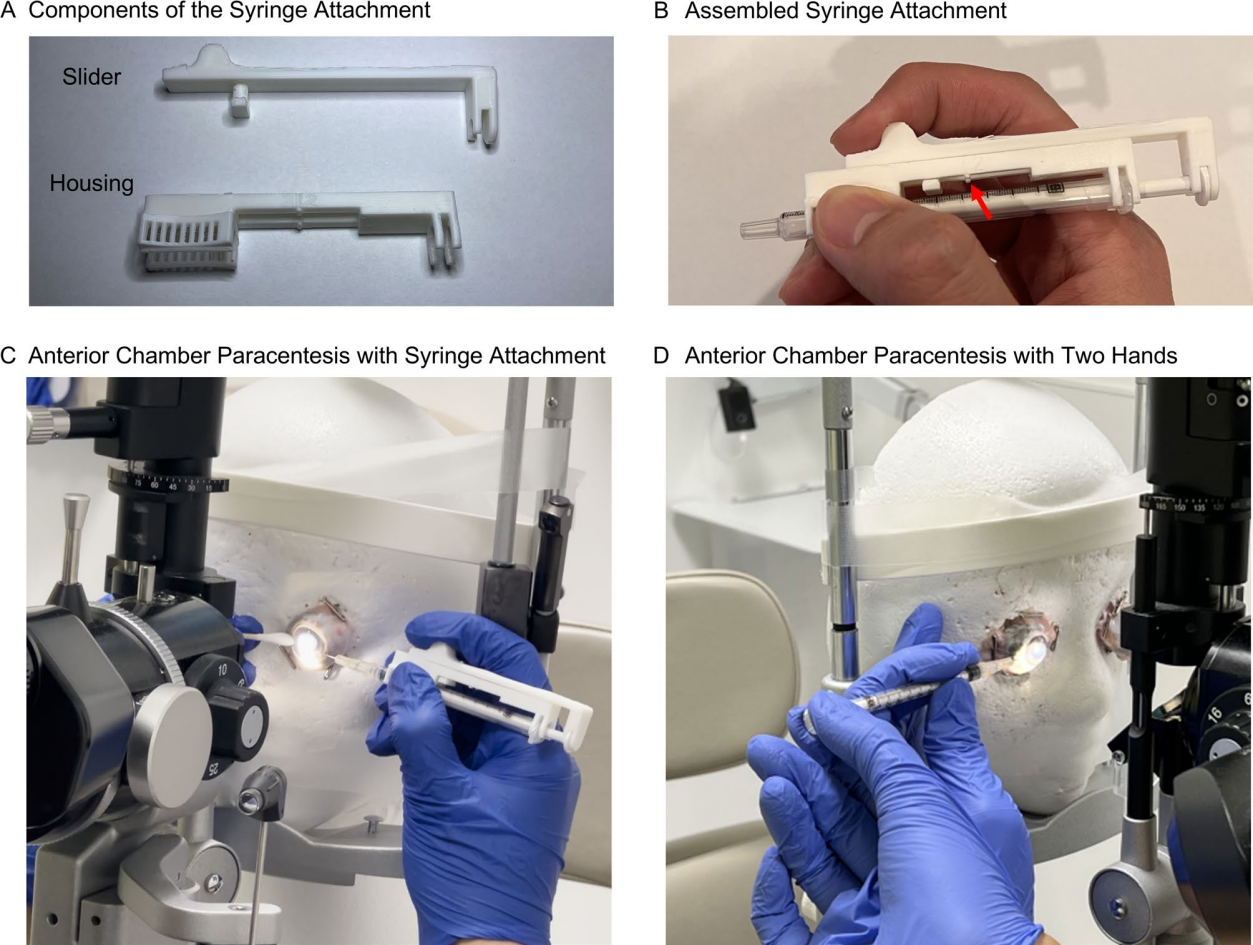
Demonstration of the novel syringe handle for anterior chamber paracentesis. (**A**) The 3D-printed syringe handle comprises a slider and a housing. (**B**) The assembled syringe handle features a hard stop at the 0.2 mL syringe mark (red arrow), preventing the extraction of more than 0.2 mL of aqueous fluid. (**C**) A photograph illustrating the syringe handle in use during anterior chamber paracentesis, where the user performs one-handed paracentesis with the right hand while stabilizing the eye using a cotton-tip applicator in the left hand. (**D**) The two-handed anterior chamber paracentesis technique used in the wet lab study, where one hand holds the syringe barrel while the other pulls the plunger

We designed the syringe handle using Autodesk Fusion 360 (Autodesk Inc., San Francisco, CA, USA) and 3D printed it with polylactic acid material. The 7-gram handle consists of a slider and a housing (Fig. 1A), with an estimated material cost of $0.14. Once assembled, the slider locks into the housing, which snaps onto a a 1-mL BD^®^ Tuberculin Syringe (Becton, Dickinson and Company, Franklin Lakes, NJ, USA) (Fig. 1B). A hard stop at the 0.2-mL syringe marker prevents over-extraction. We also created a one-piece version with the same function (eFigure 1). We uploaded the models to the National Institutes of Health 3D model repository (3DPX ID: 022172; https://3d.nih.gov/entries/3DPX-022172).

### Wet-lab study design and participants

A pilot randomized controlled wet-lab study was conducted using porcine eyes under institutional review board exemption from the University of California, San Francisco (reference number: 24-42297; clinical trial number: not applicable). Ten ophthalmology residents were recruited with informed consent. Each performed four AC paracentesis procedures—two with the novel device and two with the conventional method—for a total of 40 procedures. To reduce bias, participants were randomly assigned in a 1:1 ratio to start with either the novel or conventional method.

Fresh porcine eyes were mounted in Styrofoam head models secured to a slit lamp stand (Fig. 1C and D). Prior to each procedure, the anterior chamber was inflated with balanced salt solution. A 30-gauge needle attached to a 1-mL BD^®^ Syringe was used in all procedures. Each participant first performed the procedure on the left eye with the right hand, then on the right eye with the left hand.

In the novel method, participants held the syringe in one hand and retracted the plunger with their index finger until it reached the 0.2 mL stop, stabilizing the globe with the other hand using a cotton-tipped applicator (Fig. 1C; Supplemental Video). In the conventional method, one hand held the syringe while the other retracted the plunger (Fig. 1D). In both methods, participants were instructed to aspirate ≥ 100 µL of aqueous humor while avoiding intraocular structures. An observer (MAC or MEM, evenly split between sessions), under the supervision of LLS, prepared the wet lab setup, recorded the amount of aqueous humor extracted, procedure time, and any intraocular tissue contact, and ensured adherence to the study protocol for all participants.

### Statistical analysis

The primary outcome was success rate, defined as aspiration of ≥ 100 µL. Secondary outcomes included intraocular contact, aspirated volume, and procedure time. Participants completed an five-question anonymous post-procedure survey (Supplemental Methods). Data were analyzed in R 4.0.4 (R Foundation for Statistical Computing). We performed a linear mixed-effects model, including procedure method and eye laterality as fixed effects and participant as a random effect.

## Results

All 40 procedures achieved ≥ 100 µL fluid aspiration. No intraocular injuries occurred with the novel method. Two contacts occurred in the conventional group: one with the corneal endothelium and another involving both iris and cornea. Significant needle movement was also observed when participants looked away from the slit lamp to check volume.

**Fig. 2:**
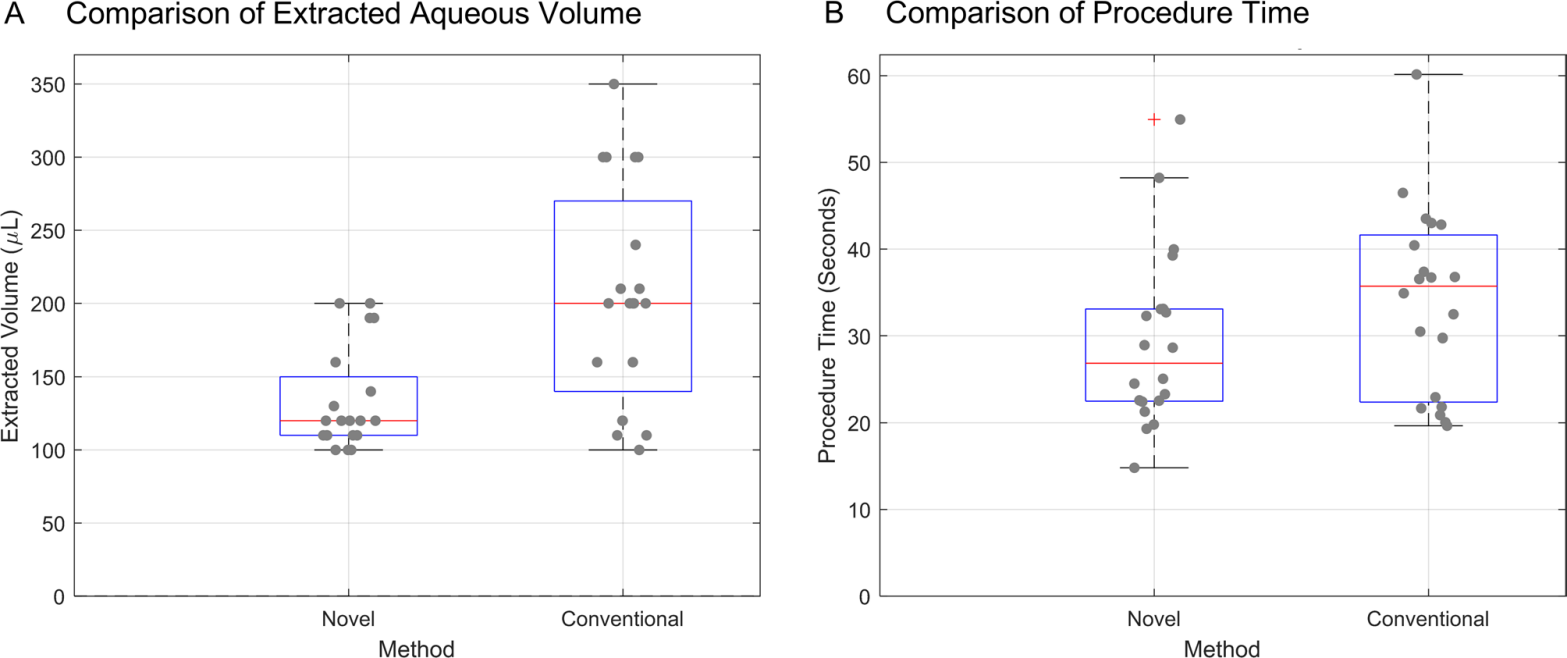
Wet lab study results comparing the novel syringe handle to the conventional two-handed anterior chamber paracentesis technique (40 procedures, 10 participants). **A**, Boxplot of extracted aqueous volume. All participants successfully extracted at least 100 µL using both methods. The novel handle had a median [interquartile range (IQR)] of 120 [110–150] µL, close to the 100 µL target, with no extractions exceeding 200 µL. In contrast, the conventional method yielded significantly higher volumes (200 [140–270] µL, *P* < 0.001), with 25% of procedures exceeding 250 µL. **B**, Boxplot comparing procedure time between the two methods. The median and IQR for the novel method was 26.9 [22.5–33.1] seconds, while the conventional method took 35.7 [22.3–41.6] seconds (*P* = 0.13)

With the novel device, the median aspirated volume was 120 µL (interquartile range [IQR]: 110–150 µL), close to the 100 µL threshold, with no extractions exceeding 200 µL. In contrast, the conventional method aspirated a median of 200 µL (IQR 140–270 µL), significantly higher than the novel method (*P* < 0.001; Fig. 2A). Five (25%) of 20 conventional procedures extracted > 250 µL.

The median procedure time was 26.9 (IQR 22.5–33.1) seconds with the novel device versus 35.7 (IQR 22.3–41.6) seconds with the conventional method (Fig. 2B), a median reduction of 8.8 s (25% reduction), though not statistically significant (*P* = 0.13).

**Fig. 3:**
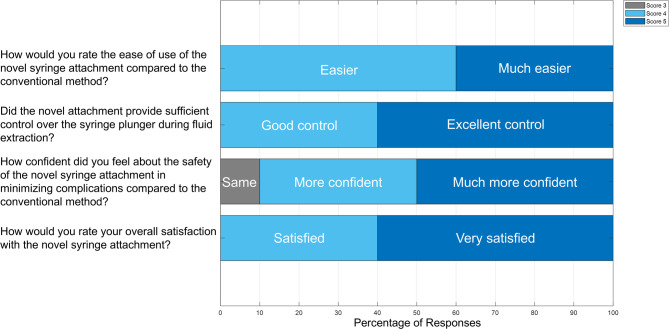
Results of the anonymous survey from 10 participants after the wet lab study. Participants rated each question on a scale from 1 to 5, with 5 indicating the most favorable response toward the novel syringe handle. All responses ranged from 3 to 5. Each bar represents aggregated responses to post-procedure usability and preference questions

Figure 3 presents the post-procedure survey results. All 10 participants rated the novel device between 3 and 5 (with 5 indicating the greatest preference towards the novel method) for ease of use, control, safety, and satisfaction. Nine of 10 reported feeling more confident about safety with the novel device, and 7 of 10 said it would likely reduce their procedural time.

## Discussion

In this study, we developed a novel syringe handle that works with standard 1-mL syringes for one-handed AC paracentesis, which improved safety and precision compared to the conventional method while reducing procedural time in a randomized controlled wet-lab study. In all 20 uses, participants successfully extracted at least 100 µL of aqueous humor by retracting the plunger to the hard stop at 0.2 mL, without looking away from the slit lamp. This improves control and safety by eliminating the need to monitor volume mid-procedure, unlike the conventional two-handed technique. The anonymous post-study survey demonstrated participants’ preference towards the novel device.

Our device may benefit other ophthalmic and medical procedures requiring aspiration or injection. In ophthalmology, it may assist with intracameral gas injection at the slit lamp [9]. It could also be applied to large-volume aspiration or injection in non-ophthalmic procedures such as joint aspiration, abscess drainage, or epidural injections—situations where one hand operates the syringe while the other stabilizes the site. Future studies should investigate these potential applications.

A limitation of this study is the small sample size and the inclusion of only resident physicians, who are still in training. While this limits the generalizability of the findings to experienced clinicians, it is suitable for evaluating the feasibility of a new device intended to assist less experienced users. This pilot study included only ophthalmology residents to standardize participant skill levels. Future studies should include both residents and attending ophthalmologists to assess whether the device offers differential benefits across varying levels of surgical experience. Another limitation is that observers were not masked to the procedure type, as participants performed both techniques—with and without the syringe handle—which were visibly distinct. Additional limitations include the single-center design and use of porcine eyes, which do not mimic human eye movement.

## Conclusions

We developed a novel syringe handle that enhances the safety and accuracy of AC paracentesis without compromising efficiency. Its simple design and compatibility with standard syringes make it a practical tool with potential to enhance the procedure. Further studies may explore broader medical applications.

## Supplementary Information

Below is the link to the electronic supplementary material.

Supplementary Material 1

Supplementary Material 2

Supplementary Material 3

## Data Availability

The 3D printing file for the anterior chamber syringe handle is available at the National Institutes of Health 3D model repository (3DPX ID: 022172; [https://3d.nih.gov/entries/3DPX-022172] (https://3d.nih.gov/entries/3DPX-022172)). All data generated or analysed during this study are included in this published article [and its supplementary information files].

## Abbreviations

AC: Anterior Chamber
IQR: Interquartile Range
