# Supplementary material for: Novel syringe handle for one-handed anterior chamber paracentesis
Source: BMC Ophthalmol. 2025 Nov 28;25:675. doi: 10.1186/s12886-025-04503-z (PMC12661716; doi:10.1186/s12886-025-04503-z)
Supplement: Supplementary file 1 — Supplementary Material 1 [file 12886_2025_4503_MOESM1_ESM.pdf]

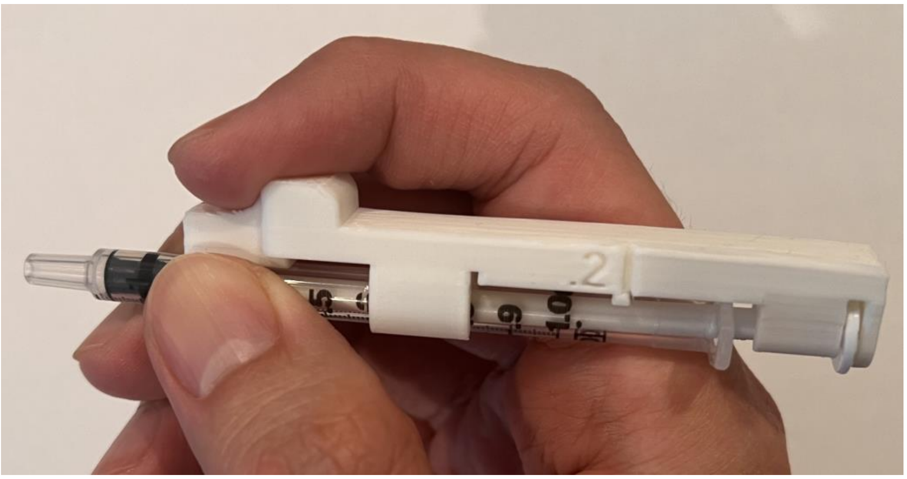

Figure S1. One-piece design of the 3D printed syringe attachment. The syringe attachment feature a hard stop at the 0.2 mL syringe mark, preventing the extraction of more than 0.2 mL of aqueous fluid.
