# Supplementary material for: Novel syringe handle for one-handed anterior chamber paracentesis
Source: BMC Ophthalmol. 2025 Nov 28;25:675. doi: 10.1186/s12886-025-04503-z (PMC12661716; doi:10.1186/s12886-025-04503-z)
Supplement: Supplementary file 2 — Supplementary Material 2 [file 12886_2025_4503_MOESM2_ESM.pdf]

Supplemental Methods. Post-Procedure Survey

1. Ease of Use: How would you rate the ease of use of the novel syringe attachment compared to the conventional method?
- 1 (Much more difficult)
- 2 (More difficult)
- 3 (About the same)
- 4 (Easier)
- 5 (Much easier)

2. Control over Syringe Plunger: Did the novel attachment provide sufficient control over the syringe plunger during fluid extraction?
- 1 (No control)
- 2 (Limited control)
- 3 (Moderate control)
- 4 (Good control)
- 5 (Excellent control)

3. *Procedure Time*: Do you feel the novel attachment allows for a faster procedure time compared to the conventional method?
- Yes
- No

4. *Confidence in Safety*: How confident did you feel about the safety of the novel syringe attachment in minimizing complications compared to the conventional method?
- 1 (Much less confident than the conventional method)
- 2 (Less confident than the conventional method)
- 3 (About the same confidence as the conventional method)
- 4 (More confident than the conventional method)
- 5 (Much more confident than the conventional method)

5. How would you rate your overall satisfaction with the novel syringe attachment?
- 1 (Very dissatisfied)
- 2 (Dissatisfied)
- 3 (Neutral)
- 4 (Satisfied)
- 5 (Very satisfied)
